# Jumping Ahead with *Sleeping Beauty*: Mechanistic Insights into Cut-and-Paste Transposition

**DOI:** 10.3390/v13010076

**Published:** 2021-01-08

**Authors:** Matthias T. Ochmann, Zoltán Ivics

**Affiliations:** Division of Medical Biotechnology, Paul Ehrlich Institute, 63225 Langen, Germany; matthias.ochmann@pei.de

**Keywords:** transposon, strand transfer, excision, synaptic complex, DNA repair, integration, DNA binding, crystal structure, transposase, DNA recombination

## Abstract

*Sleeping Beauty* (SB) is a transposon system that has been widely used as a genetic engineering tool. Central to the development of any transposon as a research tool is the ability to integrate a foreign piece of DNA into the cellular genome. Driven by the need for efficient transposon-based gene vector systems, extensive studies have largely elucidated the molecular actors and actions taking place during SB transposition. Close transposon relatives and other recombination enzymes, including retroviral integrases, have served as useful models to infer functional information relevant to SB. Recently obtained structural data on the SB transposase enable a direct insight into the workings of this enzyme. These efforts cumulatively allowed the development of novel variants of SB that offer advanced possibilities for genetic engineering due to their hyperactivity, integration deficiency, or targeting capacity. However, many aspects of the process of transposition remain poorly understood and require further investigation. We anticipate that continued investigations into the structure–function relationships of SB transposition will enable the development of new generations of transposition-based vector systems, thereby facilitating the use of SB in preclinical studies and clinical trials.

## 1. Introduction

The capacity of nucleic acids to move around and integrate into a new locus has evolved in manifold ways. Different enzymes have gained the capacity to process nucleic acids and integrate them—namely retroviruses [1,2], endogenous retroviruses [3], and homologous recombination repair mechanisms [4]. Among them, the large family of transposons first described by Barbara McClintock in maize [5] have the ability to move their genetic information within the genome. 

Transposable elements (TEs) can be classified into two groups according to their mechanism of movement. Class I TEs, also called retrotransposons, follow a copy-and-paste mechanism. After transcription of their DNA genome to RNA, a reverse transcription step back into DNA is performed, and a reintegration into the genome occurs [6]. This process has certain similarities with retroviruses. Class I retrotransposons can be further subdivided into long terminal repeat (LTR) retrotransposons; retroviruses [1,2] and endogenous retroviruses (ERV) [3]; and non-LTR retrotransposons, including long interspersed nuclear elements (LINEs, such as the L1 element [7]) and short interspersed nuclear elements (SINEs, such as the *Alu* element [8]). 

Class II transposons are DNA transposons solely relying on DNA intermediates in their transposition process. They can be subdivided into two subclasses. Subclass I follows a cut-and-paste mechanism, during which the transposon is excised from one genomic location and reintegrates somewhere else [6]. In contrast, Subclass II transposons, such as members of the *Helitron* superfamily [9], follow a copy-and-paste mechanism, during which the element generates copies of itself which integrate into the genome. However, unlike with retrotransposons, the copying mechanism does not involve an RNA intermediate. Subclass I DNA transposons include the superfamilies *Transib* [10], *piggyBac* [11], PIF/*Harbinger* [12], and Tc1/*mariner* [13]. 

All the members of the Tc1/*mariner* superfamily have in common that these elements are flanked by terminal inverted repeats (TIRs), and contain a gene encoding a transposase, an enzymatic factor catalyzing the transposition reaction [6]. The transposase binds to the TIRs, excises the transposon from the donor locus, and reintegrates it adjacent to a TA target sequence, leading to a TA target site duplication [6]. Members of the Tc1/*mariner* family are ubiquitous in eukaryotes [6]. 

Because the TIRs and the transposase are considered to constitute the minimally required components for the transposition reaction, a transposon that contains all these elements is therefore considered autonomous [14]. However, many autonomous TEs have given rise to non-autonomous derivatives by mutations, insertions, or deletions in their transposase coding regions. These non-autonomous TEs can still be mobilized, but need a functional transposase expressed by another element in the same cell [14]. It is this *trans*-complementarity between two functional components (the transposase and the specific TIRs that are recognized and mobilized by the transposase) that serves as the basis of turning transposons into genetic vector systems suitable for moving any gene of interest into the genome of a host cell. The *Sleeping Beauty* (SB) transposon system [14] is widely used as a genetic engineering tool (recently reviewed in Amberger et al. [15]). The structural features and mechanistic steps and processes taking place in the life cycle of SB from DNA binding up to integration are described in the following sections.

## 2. Structural Features of the *Sleeping Beauty* Transposon System

### 2.1. The Sleeping Beauty Transposase

**Figure 1:**
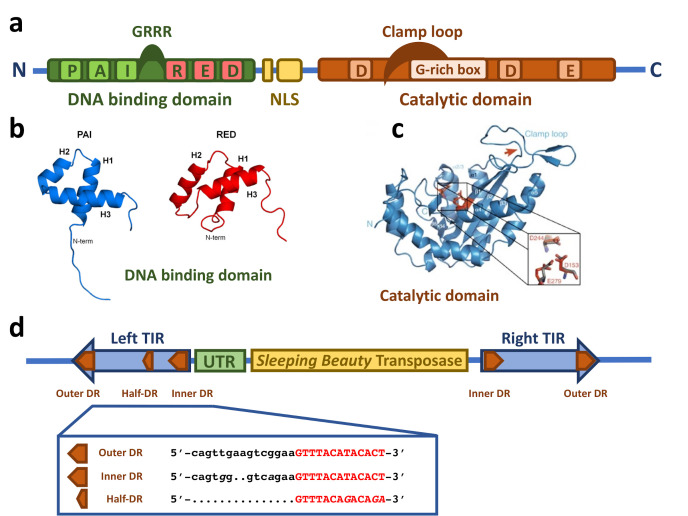
Structural features of the *Sleeping Beauty* transposable element. (**a**) Schematic drawing of the domain structure of the SB transposase. The SB transposase has an N-terminal bipartite, paired-like DNA binding domain (green box) with the helix-turn-helix PAI subdomain (light green box) and RED subdomain (red box) and a GRRR AT-hook motif. It is followed by a bipartite nuclear localization signal (NLS, yellow boxes) and a C-terminal catalytic domain (orange box), with the DDE amino acid triad catalyzing the DNA cleavage and joining reactions. The clamp loop important for protein–protein interactions is overlapping with a glycine-rich box (light orange box). (**b**) NMR structure of the PAI and RED subdomains of the SB transposase. Reprinted from *Protein Science* [21] with permission from the publisher. (**c**) Crystal structure of the catalytic domain of the SB transposase with the catalytic triad (DDE) and the clamp loop. Reprinted from *Nature Communications* [23] with permission from the publisher. (**d**) Schematic drawing of the autonomous SB transposable element with the transposase coding region (yellow box) and the TIRs (blue arrows). An untranslated region (UTR, green box) is situated between the left TIR and the transposase coding region. The TIRs contain two binding sites for the transposase (orange arrows) represented by short directs repeats (DRs), one inner and one outer DR per TIR. In addition, the left TIR contains a “half-DR” sharing sequence similarities with the DRs. The DR core sequence, with which the PAI subdomain of the SB transposase interacts, is typed in red.

The SB transposase (Figure 1a) is composed of an N-terminal DNA binding domain (DBD) (amino acids (aa) 1–110) and a C-terminal catalytic domain (DDE) (aa 114–340) connected by a flexible linker region harboring a nuclear localization signal (NLS) (aa 97–123) [14]. The DBD consists of the two subdomains PAI and RED (PAIRED-like DBD) connected by a linker [14,16]. Each subdomain is predicted to consist of three α-helices forming a helix-turn-helix (HTH) motif which is found in many DNA binding proteins [17,18,19,20]. The predicted HTH motif was confirmed by the NMR structure of the DBD subdomains [21,22] (Figure 1b). The NMR structure shows that the three helices of the PAI subdomain are located in the residues aa 12–22, aa 29–33, and aa 39–55, which are tightly packed. The HTH motif is between the second and third helices [22]. Around 30% of the PAI subdomain consists of positively charged amino acids, mainly arginines and lysines, leading to electrostatic repulsion and the destabilization of the structure in the presence of physiological salt concentration and the absence of the TIRs [22]. The three helices of the RED subdomain are located in the residues aa 67–77, aa 84–93, and aa 100–109 [21]. Helices 1 and 2 pack against each other in an antiparallel arrangement, whereas helix 3 is located on top of them [21]. The HTH motif is between helices 2 and 3; however, in contrast to the PAI subdomain, it does not show a canonical β-turn connecting both helices, but a variation in the β-turn with a longer turn-motif [21]. Additionally, helix 3 in the PAI subdomain is one turn longer [21]. Similarly to the PAI subdomain, the RED subdomain is highly positively charged, enhancing its DNA binding [21]. 

The catalytic domain is predicted to have an RNaseH-like fold, similar to other DDE recombinases [24,25]. The catalytic triad of three acidic residues (DDE) [14], giving the domain its name, catalyze the DNA hydrolysis, required for excision, and transesterification, taking place in the integration reaction, in a two-metal-ion-dependent manner [26,27]. Crystallographic structure analysis revealed the predicted RNaseH-like fold, consisting of a central five-stranded β-sheet surrounded by five α-helices [23] (Figure 1c). The three catalytic residues (D153, D244, and E279) are in close proximity, making up the active site of the enzyme [23]. The clamp loop (aa 159–190) between β1 and β2 includes a glycine-rich strip (aa 183–190) [14] which is curved and pivots on three consecutive glycines (aa 188–190) leading to an extended protein-protein surface [23]. The tip of the clamp loop has two short antiparallel β-strands (aa 169–174 and aa 174–176), forming a β-hairpin which is important for the protein–protein interaction with the inter-domain linker (aa 119–122) of a partner SB transposase molecule [23].

### 2.2. The Sleeping Beauty Transposable Element

In addition to the transposase, the TIRs of the SB transposon flanking both ends (Figure 1d) are also critically required for the transposition process. When SB is used as a gene delivery tool, any genetic cargo can be placed between the TIRs and mobilized by the transposase. The TIRs are ~220 bp in length and contain two direct repeats (DRs), one outer and one inner, serving as binding sites for the SB transposase. This TIR arrangement has been called the IR/DR structure [28]. Notably, the four DRs of SB are not identical: the outer DRs are longer than the inner DRs by 2 bps (Figure 1d), and even slight variations in the DR sequences can have a severe effect on the transposition efficiency [28,29,30,31]. The left and right TIRs are not identical either; the left TIR has an extra “half-DR” element showing sequence similarities to the transposase binding site (Figure 1d), which acts as a transpositional enhancer [16]. Downstream of the left TIRs is an untranslated region (Figure 1d) that contributes to the transcriptional regulation of the transposase [32,33].

## 3. The Mechanism of *Sleeping Beauty* Transposition

### 3.1. DNA Binding of the Sleeping Beauty Transposase

**Figure 2:**
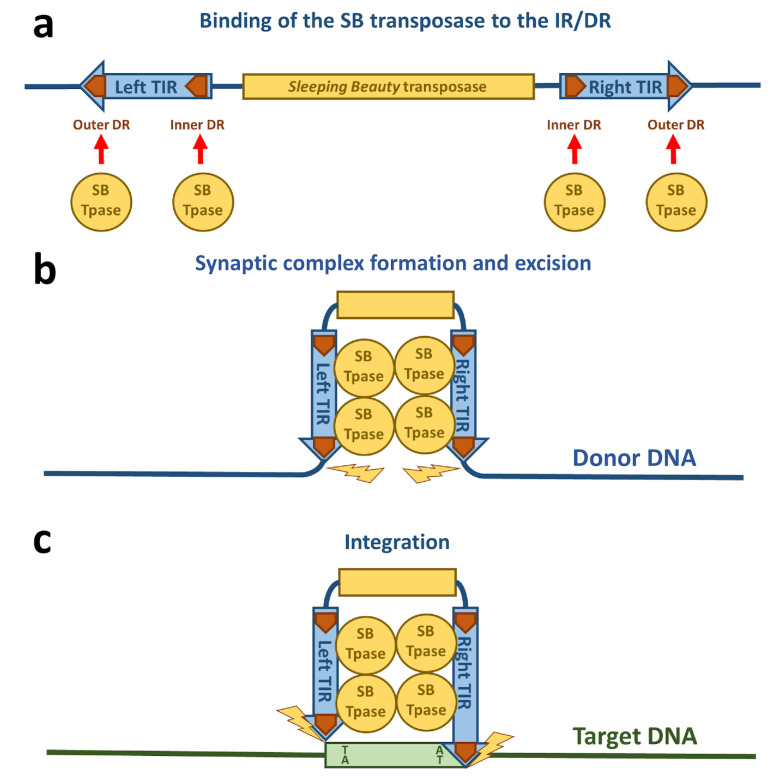
Schematic drawing of *Sleeping Beauty* transposition. (**a**) The SB transposase (blue circle) binds to the DRs (orange arrows) within the TIRs. (**b**) The TIRs are brought together by SB transposase molecules in a synaptic complex. Excision of the SB transposon takes place from the donor DNA indicated by yellow flashes. (**c**) The excised transposon integrates into a TA site in the target DNA (green box) that is afterwards duplicated and flanks the new target site.

The transposition life cycle begins with binding of the transposase to the transposon DNA (Figure 2a). The DNA binding domain of the transposase is mainly responsible for the DNA recognition. Out of the two subdomains (PAI and RED), the PAI subdomain has the dominant role in base-specific DNA binding [16]. The 3′-part of the transposase binding site containing a core sequence conserved in all four DRs is recognized by the PAI subdomain [16,22]. The DNA binding region of the PAI subdomain is located in the residues aa 28, 29, 31, 33–36, 38–43, and 47, which are situated on the second and third α-helices and on the loop connecting these helices of the HTH motif [22], which is consistent with the role of HTH motifs in DNA binding [19]. The RED subdomain interacts with the 5′-part of the DR adjacent to the core sequence [16]. This interaction of the RED subdomain with DNA occurs only in the outer DRs and not the inner DRs [22]. Residues located at the third helix of the RED subdomain have been identified to be primarily responsible for the DNA recognition of this subdomain, however helix 1 is also highly positively charged and therefore potentially capable of binding DNA [21]. All of the four transposase binding sites in the IR/DR structure in the TIRs are necessary for SB transposition [34]. An important aspect for the next steps in the life cycle of SB transposition is the formation of a transposase tetramer in a complex with the transposase binding sites [16]. The inner DRs are bound by the transpose with a higher affinity than the outer DRs [28,35], which was also confirmed by the NMR data on the PAI subdomain [22]. Additionally, the “half-DR” in the left TIR is bound by the PAI subdomain and mediates protein–protein interactions with other transposase subunits [16]. The PAI subdomain therefore fulfills three important functions: interaction with the DRs, interaction with the “half-DR”, as well as transposase oligomerization. A GRRR amino acid motif contributes as an AT-hook for specific substrate recognition [16]. In domain swapping experiments, it was shown that primary DNA binding is not sufficient to determine the specificity of the transposition reaction [16]. These experiments indicate that the RED subdomain enforces specificity at a later step in transposition and therefore prevents the mobilization of the SB transposon by transposases expressed by other, closely related subfamilies in the same genome. It was also shown that the RED subdomain is involved in protein–protein interactions and forms dimers upon DNA binding [36]. Helix 2 of the RED subdomain has neutral or negative electrostatic potential and therefore could mediate protein–protein interactions [21,36]. All these observations of the DNA-binding are consistent with the crystal structures of protein-DNA complexes of closely related Tc1/*mariner* family members such as Tc3 and Mos1 transposases [37,38]. Because the Tc3 and Mos1 transposons do not have an IR/DR-like structure of their TIRs (instead, these transposons have a single binding site for their transposases at each end of their short TIRs), the presence and strict requirement for IR/DR in SB transposition suggests a regulatory role, which is discussed in the next section.

### 3.2. Synaptic Complex Formation

**Figure 3:**
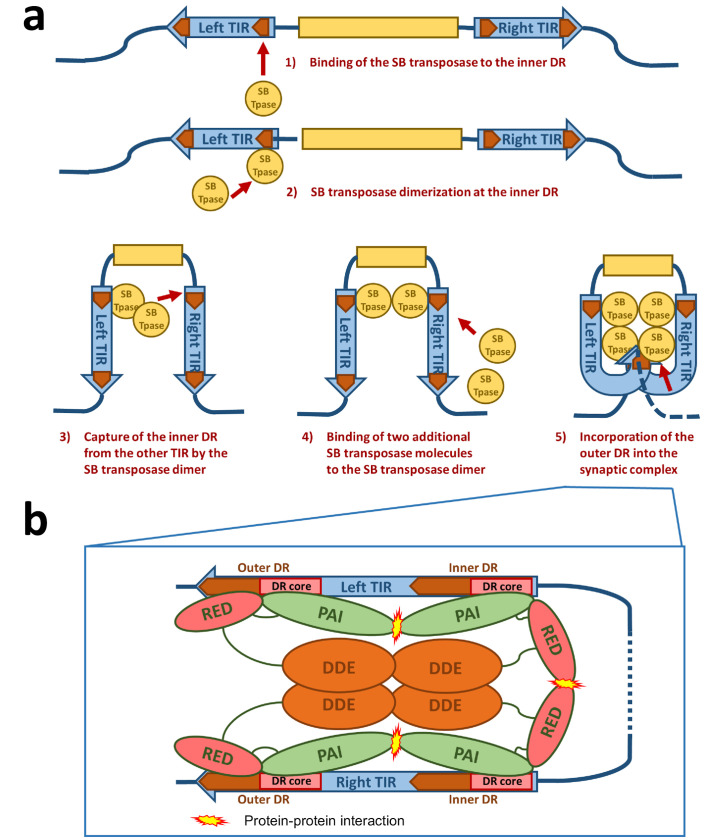
Schematic drawing of the synaptic complex formation. (**a**) At first, the SB transposase binds at the inner DR of the left TIR and forms dimers at this site. The SB dimer then captures the inner DR of the other TIR. Two additional SB transposase molecules are recruited to the nucleoprotein complex, leading to an incorporation of the outer DRs into the synaptic complex. (**b**) Protein–DNA and protein–protein interactions in the SB synaptic complex. The PAI subdomain of the N-terminal DNA-binding domain of the SB transposase interacts with the DR core sequence at both the inner and outer DRs. The RED subdomain contributes to DNA binding only at the outer DRs. At the inner DRs, the RED subdomain contributes to transposase dimerization. The relative positions of the four transposase monomers within the complex are arbitrarily drawn. Based on the structure of the Mos1 synaptic complex [37], it is likely that the catalytic DDE domains are acting in *trans*—that is, the DDE domain of an SB monomer bound at the left TIR executes cleavage at the right TIR and vice versa.

The next step required in the life cycle of SB transposition is the formation of a nucleoprotein complex called the synaptic complex (Figure 2b and Figure 3). In this complex, both ends of the transposon are paired and held together by transposase subunits. For the formation of a synaptic complex, the complete TIRs with four transposase binding sites (DRs) and tetramerization-competent SB transposase are required. The “half-DR” motif in the left TIR is not essential for transposition, but functions as an enhancer of the transposition together with the PAI subdomain. It likely stabilizes the complexes formed by a transposase tetramer bound at the TIRs [16]. 

For the formation of the synaptic complex, it has been proposed that a defined order of protein–DNA and protein–protein interactions is important [36] (Figure 3a). In this process, the assembly is mainly orchestrated by the interplay of the IR/DR structure and the PAIRED-like DNA binding domain of the SB transposase. The specific primary DNA recognition is performed by the PAI subdomain at an inner DR, which is bound at a higher affinity than the outer DRs [22,28]. The contribution of the RED subdomain to the DNA binding at the inner DR is limited, hence the transposase forms dimers through the protein–protein interaction of the RED-RED interface located in helix 2 [36]. The SB transposase could also bind to the inner DR as a preformed dimer. Once bound, this nucleoprotein complex captures the inner DR from the other TIR (Figure 3a). The incorporation of an outer DR into the synaptic complex by the transposase bound at the inner DR of the opposite TIR does not result in productive transposition. In the next step, two additional SB transposase molecules are recruited to the complex through the PAI-PAI protein interaction interface (Figure 3a,b). This leads to the incorporation of the outer DRs in the synaptic complex [36] (Figure 3a,b). In this step, the RED subdomain is required to complete the assembly process by recognizing the outer DRs, thereby preparing the complex for strand cleavage executed by the catalytic domain [36] (Figure 3b). This whole process is assisted by a host-encoded cofactor called HMGB1, which is recruited by the SB transposase to the TIRs [35]. HMGB1 facilitates DNA bending at the inner DR, which could enhance the capture of the inner DR on the other TIR [35]. However, the transposition reaction works also in the absence of HMGB1 to a lower extent [35]. This ordered assembly is an important quality control leading to functional transposition intermediates. It is important to note that if the ends of the SB transposon are too close to each other (for example, in a circular DNA molecule), the efficiency of transposition decreases [34]. Indeed, it has been established that efficient SB transposition requires at least ~300 bp DNA bridging the TIRs [34]. A possible explanation for this observation is that a certain length of DNA might be necessary to accommodate the multimeric transposases and the host factor HMGB1 during the formation of the synaptic complex. This orchestrated assembly of the synaptic complex shows that an alteration in the DNA binding affinity of the SB transposase to the DRs does not necessarily enhance the transposition reaction as a whole. Indeed, the replacement of the outer DR with the sequence from the inner DR leads to insufficient SB transposition [28]. The ordered assembly functions therefore as a “built-in” regulatory checkpoint mechanism, enforcing synaptic complex formation before excision and ensuring that DNA cleavage occurs only at the outer DRs, thereby leading to a higher level of accuracy and fidelity in contrast to other transposons with simply structured TIRs [35,39,40]. 

It is notable that the mechanistic assembly of synaptic complexes is analogous between SB transposition and V(D)J recombination. The sequences recognized by the RAG1/2 recombinase are related and binding is assisted by HMGB1 [41,42,43]. The regulation of an ordered assembly of nucleoprotein complexes by somewhat dissimilar recombination sites is also seen in V(D)J recombination [44], except that V(D)J recombination occurs between heterologous partner sites (following the so-called 12/23 rule), whereas SB transposition involves homologous sequences. 

### 3.3. Excision of the Sleeping Beauty Transposon

**Figure 4:**
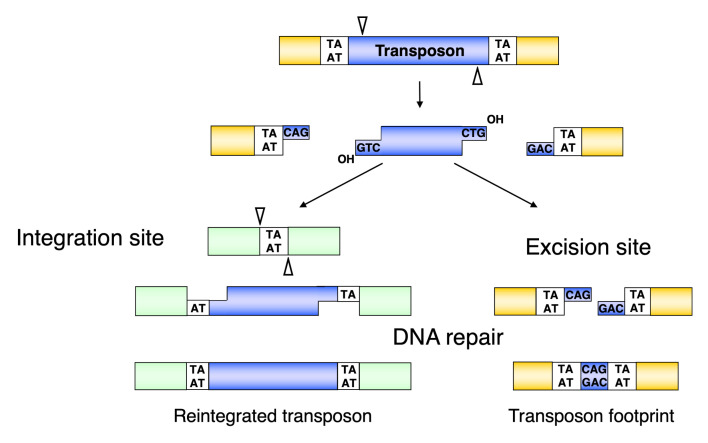
Molecular events leading towards the formation of transposon footprints and target site duplications in *Sleeping Beauty* transposition. The SB transposase excises the transposon with staggered cuts and reintegrates it at a TA target dinucleotide. The single-stranded gaps at the integration site and the double-strand DNA breaks at the donor DNA are repaired by the host DNA repair machinery. After repair, the target TA is duplicated at the integration site, and a small footprint is left behind at the site of excision. Reprinted from *CMLS* [64] with permission from the publisher.

Following the assembly of the synaptic complex, the excision of the SB transposon from the donor locus occurs and DNA double-strand break (DSB) repair on the excision site takes place (Figure 2b and Figure 4). The excision step is crucial for the later integration step, because it results in the exposure of a free 3′–OH group at the transposon ends required for the strand transfer reactions taking place at the integration site [45] (Figure 4). The first catalytic step in all transposition reactions is a Mg-cation-dependent hydrolysis of the phosphodiester bond in the DNA backbone. This process is catalyzed by all DDE recombinases in a similar way [46]—namely, first strand cleavage generates a single-strand nick by a nucleophilic attack of a H_2_O molecule, resulting in a free 3′–OH group [45]. The nicking of the first strand is followed by the cleavage of the complementary DNA stand, resulting in a double-strand break (DSB) that liberates the transposon from the donor DNA. To catalyze second strand cleavage, DDE enzymes evolved versatile strategies [47]. Most DDE transposases, including *piggyBac*, Tn10, hAT, and the RAG1/2 recombinase catalyzing V(D)J recombination, use a single active site to cleave both DNA strands at one transposon end via a DNA hairpin intermediate either on the transposon end or on the flanking donor DNA [48,49,50,51,52]. However, members of the Tc1/*mariner* family do not transpose via a hairpin intermediate, indicating that double-strand cleavage is the result of two sequential hydrolysis reactions by the transposase [53,54]. Indeed, it has recently been shown that all the chemical steps of *mariner* transposition are executed by a single transposase dimer, in which one monomer performs two sequential strand cleavage and one strand transfer reactions at the same transposon end [55]. The Mos1 *mariner* transposase cleaves the non-transferred strand first [56], and we infer that the first cleavage event during SB transposition also occurs at the non-transferred strand of the SB transposon (Figure 4). The first nick introduced by the SB and *mariner* transposases occurs three nucleotides inside the element [57,58] (Figure 4), which, following second strand cleavage at the exact tip of the transposon, generates three-nucleotide-long 3′–overhangs at the ends of both the excised transposon and those of the flanking donor DNA. The DSBs can be repaired by the non-homologous end joining (NHEJ) or homologous recombination (HR) DNA repair pathways [59,60]. The dominant way to repair transposon excision sites in somatic mammalian cells is NHEJ, which leads to transposon “footprints” being identical to the 3′–overhangs left at the donor site after SB excision [54,61] (Figure 4). Factors including Ku70 and DNA-PKcs of the NHEJ pathway have been shown to be required for SB transposition, because they are key contributors to the NHEJ repair of the excision site [54]. A physical interaction of Ku70 with the SB transposase has been observed [54], suggesting the active recruitment of repair factors to transposon excision sites by the transposase. NHEJ components have also been shown to be required for efficient retroelement integration and V(D)J recombination [62,63]. However, in contrast to V(D)J recombination, HR-dependent repair at the excision site can also occur in SB transposition [54]. The interaction of different repair factors at DNA DSBs generated by DNA transposition, retroviral integration, or V(D)J recombination probably defines how mechanistically very similar processes can lead to different products. 

CpG methylation of chromosomal DNA, leading to the formation of heterochromatin, decreases the transposition activity of different transposons [65]. However, in the case of SB transposition, CpG methylation in mouse embryonic stem (ES) cells leads to an enhanced transposition activity [66]. This effect is not restricted to SB transposons but is a feature that transposons with the characteristic IR/DR structure share [67]. A possible explanation for the enhanced transposition activity upon CpG methylation could be that due to the formation of a tight chromatin structure at the donor site, the SB transposase can more efficiently bring the distant DR sites in the TIRs closely together. 

### 3.4. Integration of the Sleeping Beauty Transposon

The free 3′–OH-groups exposed at the ends of the excised transposon are essential for the integration step because they act as nucleophiles attacking the phosphodiester bond of the target DNA (Figure 2c). This reaction can be chemically defined as a transesterification reaction that results in a covalent coupling of the transposon ends to the target DNA [14]. In Tc1/*mariner* transposition, the transposon ends attack the double-stranded target DNA in staggered positions, displaced from one another by 2 bp on the opposite strands. Thus, integration of the two ends of the transposon with 3′-overhangs at staggered positions in the target DNA results in single-stranded gaps which are filled up by the DNA repair machinery [14] (Figure 4). This characteristic leads to a duplication of the target site flanking the element called target side duplication (TSD), which is commonly observed with many transposons. In the case of SB, the integration occurs at TA dinucleotides, leading to a characteristic TA TSD [68,69,70,71], although SB integration can rarely occur at non-TA target sites [68,72].

**Figure 5:**
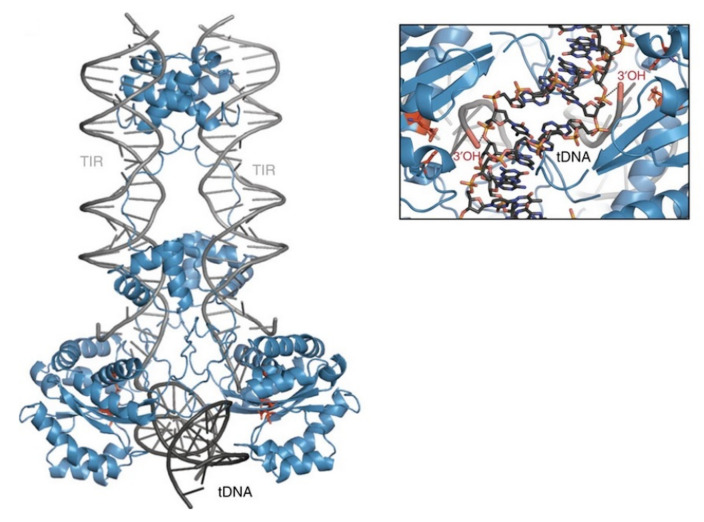
Model of the *Sleeping Beauty* strand transfer complex. Cartoon representation of the model: SB100X dimer (blue), transposon ends (TIRs, grey), and bent target DNA substrate (tDNA, dark grey). Close up of the target site showing the 3′-OH group attacking the phosphate of the TA target DNA in a staggered way. Reprinted from *Nature Communications* [23] with permission from the publisher.

Additional molecular mechanisms involved in the integration of SB remain largely unknown. However, studies on related transposases such as Mu [73] and the Tc1/*mariner* superfamily member Mos1 [37] can be related to the integration mechanism of SB. In the case of Mu transposition, the target DNA has to be bent by 140° [73]. This bend is promoted by extended interactions along the DNA backbone and by a C-terminal coiled-coil domain, reducing the electrostatic repulsion between the target DNA arms [73]. Additionally, a sharp bend of 147° was observed in the Mos1 complex [74]. It is important to note that the Mos1 post-excision complex [37] has an equivalent protein and transposon DNA arrangement, such as the strand transfer complex occurring in the integration step [74]. This implies that target DNA binding and integration occurs without major changes in the rest of the complex. Hence, the target DNA bending is important to bring the phosphate group into the active site of the preassembled transposase. This allows then the 3′-OH group of the transposon end to attack the phosphate group of the target DNA. Another important aspect of the target DNA bending is that possibly after integration at the active site the DNA snaps away, making this reaction irreversible. This product escape has been observed in different strand-transfer complexes [73,74,75,76,77]. In addition, the different spacing of the transposon ends with respect to the target DNA—which in the case of Tc1/*mariner* transposases a TA dinucleotide pair—requires a different degree of target DNA bending. It is therefore expected that the SB transposase, such as Mos1, should be equipped with the ability to severely deform the DNA double helix at >140°. Furthermore, it is likely that certain sequence-specific features at integration sites contribute to target DNA bending. Alternating pyrimidine-purine bases, known to be associated with bendable DNA structures, are often enriched in the insertion sites of most transposases and integrases [74,78]. Biochemical studies have indeed shown that flexible, bent, or mismatched sites are more suitable targets for integration [79,80,81,82]. The model of the SB target capture complex also revealed that only bent target DNA can fulfill the requirement for staggered integration [23] (Figure 5). Although the integration pattern of SB on the genome level is close to random [71], a direct interaction with the conserved TA target site has to occur. Additionally, the Mos1 strand transfer complex structure can serve here as a model for SB transposition, because it revealed a direct interaction with the adenine in the conserved TA target dinucleotide [74]. The structure shows that the adenine flips out into the extra-helical space and forms base-specific contacts with a valine (V214) of the transposase. The deformed DNA backbone is stabilized by salt bridges and hydrogen bonds with the transposase. 

Although 75% of SB transposon excision events are coupled to chromosomal integration, there is a loss of 25% of the events, which are not detectable as extrachromosomal molecules [61]. A possible explanation for this is the suicidal autointegration of the transposon into itself. This suicidal autointegration has been observed in the SB transposon [40] but also in other transposons such as Tn10 [83] or Mu [84]. The efficacy of transposition usually negatively correlates with the increasing size of the transposon [34,68,85,86,87]. One possible explanation for this drop in efficacy is the increased numbers of target sites within the transposon itself, which can lead to a higher frequency of autointegration [40]. A host factor called barrier-to-autointegration factor (BAF or BANF1) that has been identified to protect retroviruses [88,89,90,91] from autointegration was shown to interact with the SB transposase in human cells and found to inhibit the autointegration of SB [40]. 

The molecular mechanisms involved in SB transposition also have a dramatic impact on the distribution of integrations across the genome. Indeed, although SB integration is close to random over the genome when transposition is launched out of extrachromosomal plasmids [71], target site distribution is fundamentally different when the SB transposon is mobilized out of a chromosomal site. When mobilized from a chromosome, an effect called “local hopping” can be observed. Local hopping is a phenomenon where transposition out of a chromosome leads to preferred integration into *cis*-linked sites in the close vicinity of the donor locus. This feature seems to be shared by all transposons following the cut-and-paste mechanism, but the extent of this effect varies between different transposons. In the case of the P-element transposon from *Drosophila*, the rate to insert within a window of 100 kb from the donor site is ~50-fold higher than in regions outside this window [92]. Chromosomal SB transposition results in 30–80% of re-integrations occurring locally [61,93,94,95,96,97,98,99], but in a larger (up to 15 Mb) window around the donor site [98,100,101]. The extent of local hopping is not only divergent between different transposons but is also dependent on the host genome and the donor locus itself [102]. The underlying mechanism of this effect remains unknown, but a potential explanation could be varying affinities of the transposase for chromatin-associated factors in different hosts and locations within the chromosome or the instability of the post-excision complex itself, which could limit the diffusion of the complex away from the donor locus. 

## 4. New *Sleeping Beauty* Variants Offering New Possibilities for Genetic Engineering

### 4.1. Hyperactive Sleeping Beauty Transposase Variants

**Figure 6:**
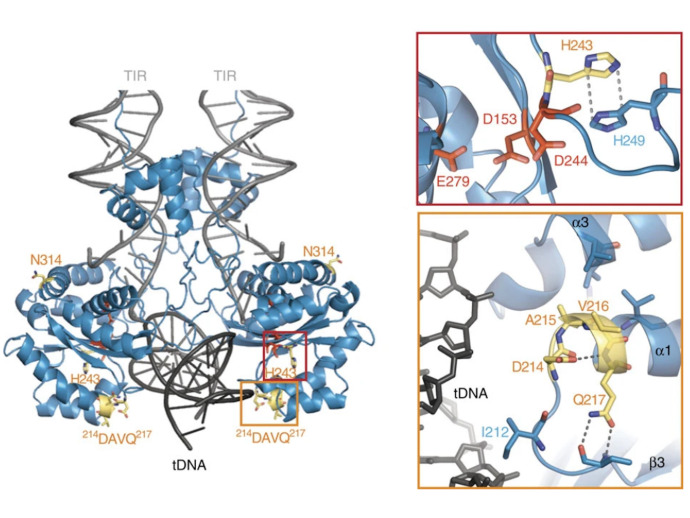
Rationalizing the hyperactive mutations of SB100X. The DAVQ (aa 214–217) mutations (highlighted in yellow box) target the binding groove of the target DNA (tDNA). The H243 mutation (highlighted in red box) together with H249 helps to position D244 in the active site of the transposase. Reprinted from *Nature Communications* [23] with permission from the publisher.

SB was reconstructed from non-autonomous Tc1 family transposons in fish genomes [14], and continued efforts to increase the transposition activity of the SB transposase have identified several mutations that lead to an overall higher integration efficiency. These mutations culminated in a hyperactive transposase variant of SB called SB100X [103]. This variant has a 100-fold increased integration efficiency compared to the first-generation SB transposase. SB100X was generated by molecular evolution and a combination of different mutants. The mutations present in this hyperactive variant were rationalized by the resolved crystal structure of the catalytic domain [23] (Figure 6). The T314N mutation in SB100X may aid the proper folding of the transposase, which has been shown to be a limiting factor in transposition [23,103]. M243H is located next to the catalytic residue D244 and forms together with H249 a π-stack, which helps to position D244 in the active site of the transposase [23]. The RKEN214—217DAVQ mutations form a part of the target binding groove, so it is likely that these mutations lead to an ideal positioning of this β3-α1 linker for an interaction with the transposon DNA [23]. By understanding the structure and mechanism of SB transposition, further hyperactive mutations could be rationally designed in the future—for example, to address the need for the efficient chromosomal integration of large SB transposons, which otherwise tend to transpose less efficiently than shorter ones [34]. Having new hyperactive variants could facilitate the integration of even larger DNA fragments over 100 kb [104] in gene therapy.

### 4.2. New Vector Platforms for Sleeping Beauty Transposition

The generation and use of new hyperactive SB transposase variants can not only increase integration efficiency. The vector platform—i.e., the DNA molecules from which transposition is initiated—can also have significant effects on the transposition efficiency. Because, as described above in the context of excision, the TIRs of the SB transposon need to be brought closely together for the catalytic steps to commence, it is likely that derivatives of circular plasmid vectors with minimal DNA sequences connecting the transposon TIRs could enhance this step. Indeed, the use of minicircles (circular genes derived from plasmids lacking bacterial backbone sequences [105]) enabled a ~20-fold increase in transposition efficiency as compared to conventional plasmids [106]. With SB minicircles, the cellular toxicity triggered by the electroporation of naked DNA was reduced up to 50% as compared to plasmid DNA in human CD34^+^ cells [106]. By delivering the SB transposase in the form of mRNA instead of an expression plasmid, the transposition efficiency was further increased and biosafety was improved due to the limited time of SB transposase present in target cells [106]. The state of the art in clinical trials is to deliver the components for SB transposition as minicircle DNA for the transposon and mRNA for the transposase [107].

### 4.3. Integration-Deficient Sleeping Beauty Transposase Variant

An interesting variant of the SB transposase which has recently been described is the K248T mutant [108]. This mutant is competent in transposon excision but has the feature of deficiency in transposon integration [108]. After excision by K248T, extrachromosomal transposon circles are formed which apparently cannot undergo integration. A structural model indicates that K248 is involved in interaction with the target DNA [108]. It is thus likely that the K248T mutant impairs the interaction with the target DNA, resulting in this integration-deficient transposase variant. This variant has been used for the generation of reprogramming factor-free induced pluripotent stem cells [108].

### 4.4. Targeted Sleeping Beauty Transposition

Targeting the integration of transposons into defined genomic regions has been challenging. However, several attempts have been made to fuse DNA binding domains to transposases, thereby targeting integration in the vicinity of sites specified by the DNA binding domains [109]. These attempts resulted in a low efficiency of targeted transposition [70] or worked only in an artificial *in vitro* environment [93] or in inter-plasmid settings [110,111]. Targeted transposition was successful in a bacterial context [112,113], but still remains challenging in eukaryotic cells. By using the CRISPR/Cas9 system with its high targeting efficiency [114], SB100X transposase fused with catalytically inactive Cas9 (dCas9), and single guide RNAs (sgRNAs) targeting the human *Alu* retrotransposon, a slight bias towards integration with *Alu* elements could be accomplished in human cells [115]. However, the efficiency of the targeted transposition events remained low and further studies are required to bias SB integrations towards defined target regions. 

## 5. Conclusions

In this review, we described the structural features and mechanistic steps involved in SB transposition. In the last few years, new structural information on the domains of the SB transposase has provided new insight into important regions, motifs, and amino acids required for transposition [21,22,23]. We have also discussed the key mechanistic steps taking place in SB transposition, from DNA binding and synaptic complex formation to SB transposon excision and up to integration. However, certain mechanistic steps—for example, during transposon integration—are not well understood in SB transposition, and need further investigation. Well-studied transposon relatives such as Mos1 offer models from which we can infer information for SB transposition [74]. However, structural features also revealed differences within the transposase structure. To gain further understanding of the underlying structure-function relationships that are relevant in the context of a transposition reaction, the full-length SB transposase as present in a synaptic complex and/or strand transfer complex needs to be analyzed. Our state-of-the-art comprehension of SB transposition yielded new variants, such as hyperactive transposase variants [103], new vector systems based on minicircles [106], and integration-deficient SB transposase variants [108]. The further and deeper understanding of SB transposition could facilitate the generation of new variants, facilitating the development of an even richer SB transposon toolbox.

## Data Availability

Readers are encouraged to consult the primary research articles cited in this review article for accessing research data.

## References

[1] Seelamgari A., Maddukuri A., Berro R., de La Fuente C., Kehn K., Deng L., Dadgar S., Bottazzi M.E., Ghedin E., Pumfery A. (2004). Role of viral regulatory and accessory proteins in HIV-1 replication. Front. Biosci..

[2] Frankel A.D., Young J.A. (1998). HIV-1: Fifteen proteins and an RNA. Annu. Rev. Biochem..

[3] Bannert N., Kurth R. (2006). The evolutionary dynamics of human endogenous retroviral families. Annu. Rev. Genom. Hum. Genet..

[4] Jasin M., Rothstein R. (2013). Repair of strand breaks by homologous recombination. Cold Spring Harb. Perspect. Biol..

[5] McClintock B. (1953). Induction of Instability at Selected Loci in Maize. Genetics.

[6] Wicker T., Sabot F., Hua-Van A., Bennetzen J.L., Capy P., Chalhoub B., Flavell A., Leroy P., Morgante M., Panaud O. (2007). A unified classification system for eukaryotic transposable elements. Nat. Rev. Genet..

[7] Beck C.R., Garcia-Perez J.L., Badge R.M., Moran J.V. (2011). LINE-1 elements in structural variation and disease. Annu. Rev. Genomics Hum. Genet..

[8] Deininger P. (2011). Alu elements: Know the SINEs. Genome Biol..

[9] Kapitonov V.V., Jurka J. (2005). RAG1 core and V(D)J recombination signal sequences were derived from Transib transposons. PLoS Biol..

[10] Kapitonov V.V., Jurka J. (2001). Rolling-circle transposons in eukaryotes. Proc. Natl. Acad. Sci. USA.

[11] Sarkar A., Sim C., Hong Y.S., Hogan J.R., Fraser M.J., Robertson H.M., Collins F.H. (2003). Molecular evolutionary analysis of the widespread piggyBac transposon family and related “domesticated” sequences. Mol. Genet. Genom..

[12] Jurka J., Kapitonov V.V. (2001). PIFs meet Tourists and Harbingers: A superfamily reunion. Proc. Natl. Acad. Sci. USA.

[13] Shao H., Tu Z. (2001). Expanding the diversity of the IS630-Tc1-mariner superfamily: Discovery of a unique DD37E transposon and reclassification of the DD37D and DD39D transposons. Genetics.

[14] Ivics Z., Hackett P.B., Plasterk R.H., Izsvák Z. (1997). Molecular Reconstruction of Sleeping Beauty, a Tc1-like Transposon from Fish, and Its Transposition in Human Cells. Cell.

[15] Amberger M., Ivics Z. (2020). Latest Advances for the Sleeping Beauty Transposon System: 23 Years of Insomnia but Prettier than Ever: Refinement and Recent Innovations of the Sleeping Beauty Transposon System Enabling Novel, Nonviral Genetic Engineering Applications. Bioessays.

[16] Izsvák Z., Khare D., Behlke J., Heinemann U., Plasterk R.H., Ivics Z. (2002). Involvement of a bifunctional, paired-like DNA-binding domain and a transpositional enhancer in Sleeping Beauty transposition. J. Biol. Chem..

[17] Pabo C.O., Sauer R.T. (1992). Transcription factors: Structural families and principles of DNA recognition. Annu. Rev. Biochem..

[18] Czerny T., Schaffner G., Busslinger M. (1993). DNA sequence recognition by Pax proteins: Bipartite structure of the paired domain and its binding site. Genes Dev..

[19] Brennan R.G., Matthews B.W. (1989). The helix-turn-helix DNA binding motif. J. Biol. Chem..

[20] Aravind L., Anantharaman V., Balaji S., Babu M.M., Iyer L.M. (2005). The many faces of the helix-turn-helix domain: Transcription regulation and beyond. FEMS Microbiol. Rev..

[21] Konnova T.A., Singer C.M., Nesmelova I.V. (2017). NMR solution structure of the RED subdomain of the Sleeping Beauty transposase. Protein Sci..

[22] Carpentier C.E., Schreifels J.M., Aronovich E.L., Carlson D.F., Hackett P.B., Nesmelova I.V. (2014). NMR structural analysis of Sleeping Beauty transposase binding to DNA. Protein Sci..

[23] Voigt F., Wiedemann L., Zuliani C., Querques I., Sebe A., Mátés L., Izsvák Z., Ivics Z., Barabas O. (2016). Sleeping Beauty transposase structure allows rational design of hyperactive variants for genetic engineering. Nat. Commun..

[24] Hickman A.B., Chandler M., Dyda F. (2010). Integrating prokaryotes and eukaryotes: DNA transposases in light of structure. Crit. Rev. Biochem. Mol. Biol..

[25] Rice P.A., Baker T.A. (2001). Comparative architecture of transposase and integrase complexes. Nat. Struct Biol..

[26] Montaño S.P., Rice P.A. (2011). Moving DNA around: DNA transposition and retroviral integration. Curr. Opin. Struct. Biol..

[27] Yang W., Lee J.Y., Nowotny M. (2006). Making and breaking nucleic acids: Two-Mg^2+^-ion catalysis and substrate specificity. Mol. Cell.

[28] Cui Z., Geurts A.M., Liu G., Kaufman C.D., Hackett P.B. (2002). Structure–Function Analysis of the Inverted Terminal Repeats of the Sleeping Beauty Transposon. J. Mol. Biol..

[29] Liu G., Aronovich E.L., Cui Z., Whitley C.B., Hackett P.B. (2004). Excision of Sleeping Beauty transposons: Parameters and applications to gene therapy. J. Gene Med..

[30] Zayed H., Izsvák Z., Walisko O., Ivics Z. (2004). Development of hyperactive sleeping beauty transposon vectors by mutational analysis. Mol. Ther..

[31] Geurts A.M., Yang Y., Clark K.J., Liu G., Cui Z., Dupuy A.J., Bell J.B., Largaespada D.A., Hackett P.B. (2003). Gene transfer into genomes of human cells by the sleeping beauty transposon system. Mol. Ther..

[32] Walisko O., Schorn A., Rolfs F., Devaraj A., Miskey C., Izsvák Z., Ivics Z. (2008). Transcriptional activities of the Sleeping Beauty transposon and shielding its genetic cargo with insulators. Mol. Ther..

[33] Moldt B., Yant S.R., Andersen P.R., Kay M.A., Mikkelsen J.G. (2007). Cis-acting gene regulatory activities in the terminal regions of sleeping beauty DNA transposon-based vectors. Hum. Gene Ther..

[34] Izsvák Z., Ivics Z., Plasterk R.H. (2000). Sleeping Beauty, a wide host-range transposon vector for genetic transformation in vertebrates. J. Mol. Biol..

[35] Zayed H., Izsvák Z., Khare D., Heinemann U., Ivics Z. (2003). The DNA-bending protein HMGB1 is a cellular cofactor of Sleeping Beauty transposition. Nucleic Acids Res..

[36] Wang Y., Pryputniewicz-Dobrinska D., Nagy E.É., Kaufman C.D., Singh M., Yant S., Wang J., Dalda A., Kay M.A., Ivics Z. (2017). Regulated complex assembly safeguards the fidelity of Sleeping Beauty transposition. Nucleic Acids Res..

[37] Richardson J.M., Colloms S.D., Finnegan D.J., Walkinshaw M.D. (2009). Molecular architecture of the Mos1 paired-end complex: The structural basis of DNA transposition in a eukaryote. Cell.

[38] Watkins S., van Pouderoyen G., Sixma T.K. (2004). Structural analysis of the bipartite DNA-binding domain of Tc3 transposase bound to transposon DNA. Nucleic Acids Res..

[39] Bouuaert C.C., Liu D., Chalmers R. (2011). A simple topological filter in a eukaryotic transposon as a mechanism to suppress genome instability. Mol. Cell. Biol..

[40] Wang Y., Wang J., Devaraj A., Singh M., Jimenez Orgaz A., Chen J.-X., Selbach M., Ivics Z., Izsvák Z. (2014). Suicidal autointegration of sleeping beauty and piggyBac transposons in eukaryotic cells. PLoS Genet..

[41] West R.B., Lieber M.R. (1998). The RAG-HMG1 complex enforces the 12/23 rule of V(D)J recombination specifically at the double-hairpin formation step. Mol. Cell. Biol..

[42] Van Gent D.C., Hiom K., Paull T.T., Gellert M. (1997). Stimulation of V(D)J cleavage by high mobility group proteins. EMBO J..

[43] Agrawal A., Eastman Q.M., Schatz D.G. (1998). Transposition mediated by RAG1 and RAG2 and its implications for the evolution of the immune system. Nature.

[44] Jones J.M., Gellert M. (2002). Ordered assembly of the V(D)J synaptic complex ensures accurate recombination. EMBO J..

[45] Mizuuchi K. (1992). Polynucleotidyl transfer reactions in transpositional DNA recombination. J. Biol. Chem..

[46] Craig N.L. (1995). Unity in transposition reactions. Science.

[47] Hickman A.B., Dyda F. (2015). Mechanisms of DNA Transposition. Microbiol. Spectr..

[48] Hencken C.G., Li X., Craig N.L. (2012). Functional characterization of an active Rag-like transposase. Nat. Struct. Mol. Biol..

[49] Zhou L., Mitra R., Atkinson P.W., Hickman A.B., Dyda F., Craig N.L. (2004). Transposition of hAT elements links transposable elements and V(D)J recombination. Nature.

[50] Mitra R., Fain-Thornton J., Craig N.L. (2008). piggyBac can bypass DNA synthesis during cut and paste transposition. EMBO J..

[51] Bischerour J., Chalmers R. (2009). Base flipping in tn10 transposition: An active flip and capture mechanism. PLoS ONE.

[52] Bischerour J., Lu C., Roth D.B., Chalmers R. (2009). Base flipping in V(D)J recombination: Insights into the mechanism of hairpin formation, the 12/23 rule, and the coordination of double-strand breaks. Mol. Cell. Biol..

[53] Richardson J.M., Dawson A., O’Hagan N., Taylor P., Finnegan D.J., Walkinshaw M.D. (2006). Mechanism of Mos1 transposition: Insights from structural analysis. EMBO J..

[54] Izsvák Z., Stüwe E.E., Fiedler D., Katzer A., Jeggo P.A., Ivics Z. (2004). Healing the Wounds Inflicted by Sleeping Beauty Transposition by Double-Strand Break Repair in Mammalian Somatic Cells. Mol. Cell.

[55] Claeys Bouuaert C., Chalmers R. (2017). A single active site in the mariner transposase cleaves DNA strands of opposite polarity. Nucleic Acids Res..

[56] Dawson A., Finnegan D.J. (2003). Excision of the Drosophila Mariner Transposon Mos1. Mol. Cell.

[57] Miskey C., Papp B., Mátés L., Sinzelle L., Keller H., Izsvák Z., Ivics Z. (2007). The ancient mariner sails again: Transposition of the human Hsmar1 element by a reconstructed transposase and activities of the SETMAR protein on transposon ends. Mol. Cell. Biol..

[58] Lampe D.J., Churchill M.E., Robertson H.M. (1996). A purified mariner transposase is sufficient to mediate transposition in vitro. EMBO J..

[59] Lohe A.R., Timmons C., Beerman I., Lozovskaya E.R., Hartl D.L. (2000). Self-inflicted wounds, template-directed gap repair and a recombination hotspot. Effects of the mariner transposase. Genetics.

[60] Engels W.R., Johnson-Schlitz D.M., Eggleston W.B., Sved J. (1990). High-frequency P element loss in Drosophila is homolog dependent. Cell.

[61] Luo G., Ivics Z., Izsvák Z., Bradley A. (1998). Chromosomal transposition of a Tc1/mariner-like element in mouse embryonic stem cells. Proc. Natl. Acad. Sci. USA.

[62] Daniel R., Katz R.A., Skalka A.M. (1999). A role for DNA-PK in retroviral DNA integration. Science.

[63] Jackson S.P., Jeggo P.A. (1995). DNA double-strand break repair and V(D)J recombination: Involvement of DNA-PK. Trends Biochem. Sci..

[64] Miskey C., Izsvák Z., Kawakami K., Ivics Z. (2005). DNA transposons in vertebrate functional genomics. Cell. Mol. Life Sci..

[65] Yoder J.A., Walsh C.P., Bestor T.H. (1997). Cytosine methylation and the ecology of intragenomic parasites. Trends Genet..

[66] Yusa K., Takeda J., Horie K. (2004). Enhancement of Sleeping Beauty transposition by CpG methylation: Possible role of heterochromatin formation. Mol. Cell. Biol..

[67] Jursch T., Miskey C., Izsvák Z., Ivics Z. (2013). Regulation of DNA transposition by CpG methylation and chromatin structure in human cells. Mob. DNA.

[68] Li X., Ewis H., Hice R.H., Malani N., Parker N., Zhou L., Feschotte C., Bushman F.D., Atkinson P.W., Craig N.L. (2013). A resurrected mammalian hAT transposable element and a closely related insect element are highly active in human cell culture. Proc. Natl. Acad. Sci. USA.

[69] Liu G., Geurts A.M., Yae K., Srinivasan A.R., Fahrenkrug S.C., Largaespada D.A., Takeda J., Horie K., Olson W.K., Hackett P.B. (2005). Target-site preferences of Sleeping Beauty transposons. J. Mol. Biol..

[70] Voigt K., Gogol-Döring A., Miskey C., Chen W., Cathomen T., Izsvák Z., Ivics Z. (2012). Retargeting sleeping beauty transposon insertions by engineered zinc finger DNA-binding domains. Mol. Ther..

[71] Moldt B., Miskey C., Staunstrup N.H., Gogol-Döring A., Bak R.O., Sharma N., Mátés L., Izsvák Z., Chen W., Ivics Z. (2011). Comparative genomic integration profiling of Sleeping Beauty transposons mobilized with high efficacy from integrase-defective lentiviral vectors in primary human cells. Mol. Ther..

[72] de Jong J., Akhtar W., Badhai J., Rust A.G., Rad R., Hilkens J., Berns A., van Lohuizen M., Wessels L.F.A., de Ridder J. (2014). Chromatin landscapes of retroviral and transposon integration profiles. PLoS Genet..

[73] Montaño S.P., Pigli Y.Z., Rice P.A. (2012). The μ transpososome structure sheds light on DDE recombinase evolution. Nature.

[74] Morris E.R., Grey H., McKenzie G., Jones A.C., Richardson J.M. (2016). A bend, flip and trap mechanism for transposon integration. Elife.

[75] Passos D.O., Li M., Yang R., Rebensburg S.V., Ghirlando R., Jeon Y., Shkriabai N., Kvaratskhelia M., Craigie R., Lyumkis D. (2017). Cryo-EM structures and atomic model of the HIV-1 strand transfer complex intasome. Science.

[76] Yin Z., Shi K., Banerjee S., Pandey K.K., Bera S., Grandgenett D.P., Aihara H. (2016). Crystal structure of the Rous sarcoma virus intasome. Nature.

[77] Maertens G.N., Hare S., Cherepanov P. (2010). The mechanism of retroviral integration from X-ray structures of its key intermediates. Nature.

[78] Maskell D.P., Renault L., Serrao E., Lesbats P., Matadeen R., Hare S., Lindemann D., Engelman A.N., Costa A., Cherepanov P. (2015). Structural basis for retroviral integration into nucleosomes. Nature.

[79] Yanagihara K., Mizuuchi K. (2002). Mismatch-targeted transposition of Mu: A new strategy to map genetic polymorphism. Proc. Natl. Acad. Sci. USA.

[80] Fuller J.R., Rice P.A. (2017). Target DNA bending by the Mu transpososome promotes careful transposition and prevents its reversal. Elife.

[81] Kuduvalli P.N., Rao J.E., Craig N.L. (2001). Target DNA structure plays a critical role in Tn7 transposition. EMBO J..

[82] Pribil P.A., Haniford D.B. (2003). Target DNA Bending is an Important Specificity Determinant in Target Site Selection in Tn10 Transposition. J. Mol. Biol..

[83] Benjamin H.W., Kleckner N. (1989). Intramolecular transposition by Tn10. Cell.

[84] Maxwell A., Craigie R., Mizuuchi K. (1987). B protein of bacteriophage mu is an ATPase that preferentially stimulates intermolecular DNA strand transfer. Proc. Natl. Acad. Sci. USA.

[85] Karsi A., Moav B., Hackett P., Liu Z. (2001). Effects of insert size on transposition efficiency of the sleeping beauty transposon in mouse cells. Mar. Biotechnol..

[86] Lampe D.J., Grant T.E., Robertson H.M. (1998). Factors affecting transposition of the Himar1 mariner transposon in vitro. Genetics.

[87] Fischer S.E., van Luenen H.G., Plasterk R.H. (1999). Cis requirements for transposition of Tc1-like transposons in *C. elegans*. Mol. Genet. Genom..

[88] Mansharamani M., Graham D.R.M., Monie D., Lee K.K., Hildreth J.E.K., Siliciano R.F., Wilson K.L. (2003). Barrier-to-autointegration factor BAF binds p55 Gag and matrix and is a host component of human immunodeficiency virus type 1 virions. J. Virol..

[89] Suzuki Y., Craigie R. (2002). Regulatory mechanisms by which barrier-to-autointegration factor blocks autointegration and stimulates intermolecular integration of Moloney murine leukemia virus preintegration complexes. J. Virol..

[90] Lee M.S., Craigie R. (1998). A previously unidentified host protein protects retroviral DNA from autointegration. Proc. Natl. Acad. Sci. USA.

[91] Lee M.S., Craigie R. (1994). Protection of retroviral DNA from autointegration: Involvement of a cellular factor. Proc. Natl. Acad. Sci. USA.

[92] Tower J., Karpen G.H., Craig N., Spradling A.C. (1993). Preferential transposition of Drosophila P elements to nearby chromosomal sites. Genetics.

[93] Ruf S., Symmons O., Uslu V.V., Dolle D., Hot C., Ettwiller L., Spitz F. (2011). Large-scale analysis of the regulatory architecture of the mouse genome with a transposon-associated sensor. Nat. Genet..

[94] Dupuy A.J., Fritz S., Largaespada D.A. (2001). Transposition and gene disruption in the male germline of the mouse. Genesis.

[95] Kokubu C., Horie K., Abe K., Ikeda R., Mizuno S., Uno Y., Ogiwara S., Ohtsuka M., Isotani A., Okabe M. (2009). A transposon-based chromosomal engineering method to survey a large cis-regulatory landscape in mice. Nat. Genet..

[96] Liang Q., Kong J., Stalker J., Bradley A. (2009). Chromosomal mobilization and reintegration of Sleeping Beauty and PiggyBac transposons. Genesis.

[97] Horie K., Yusa K., Yae K., Odajima J., Fischer S.E.J., Keng V.W., Hayakawa T., Mizuno S., Kondoh G., Ijiri T. (2003). Characterization of Sleeping Beauty transposition and its application to genetic screening in mice. Mol. Cell. Biol..

[98] Carlson C.M., Dupuy A.J., Fritz S., Roberg-Perez K.J., Fletcher C.F., Largaespada D.A. (2003). Transposon mutagenesis of the mouse germline. Genetics.

[99] Fischer S.E., Wienholds E., Plasterk R.H. (2001). Regulated transposition of a fish transposon in the mouse germ line. Proc. Natl. Acad. Sci. USA.

[100] Yergeau D.A., Kelley C.M., Kuliyev E., Zhu H., Johnson Hamlet M.R., Sater A.K., Wells D.E., Mead P.E. (2011). Remobilization of Sleeping Beauty transposons in the germline of Xenopus tropicalis. Mob. DNA.

[101] Keng V.W., Yae K., Hayakawa T., Mizuno S., Uno Y., Yusa K., Kokubu C., Kinoshita T., Akagi K., Jenkins N.A. (2005). Region-specific saturation germline mutagenesis in mice using the Sleeping Beauty transposon system. Nat. Methods.

[102] Knapp S., Larondelle Y., Rossberg M., Furtek D., Theres K. (1994). Transgenic tomato lines containing Ds elements at defined genomic positions as tools for targeted transposon tagging. Mol. Genet. Genom..

[103] Mátés L., Chuah M.K.L., Belay E., Jerchow B., Manoj N., Acosta-Sanchez A., Grzela D.P., Schmitt A., Becker K., Matrai J. (2009). Molecular evolution of a novel hyperactive Sleeping Beauty transposase enables robust stable gene transfer in vertebrates. Nat. Genet..

[104] Rostovskaya M., Fu J., Obst M., Baer I., Weidlich S., Wang H., Smith A.J.H., Anastassiadis K., Stewart A.F. (2012). Transposon-mediated BAC transgenesis in human ES cells. Nucleic Acids Res..

[105] Darquet A.M., Cameron B., Wils P., Scherman D., Crouzet J. (1997). A new DNA vehicle for nonviral gene delivery: Supercoiled minicircle. Gene Ther..

[106] Holstein M., Mesa-Nuñez C., Miskey C., Almarza E., Poletti V., Schmeer M., Grueso E., Ordóñez Flores J.C., Kobelt D., Walther W. (2018). Efficient Non-viral Gene Delivery into Human Hematopoietic Stem Cells by Minicircle Sleeping Beauty Transposon Vectors. Mol. Ther..

[107] Monjezi R., Miskey C., Gogishvili T., Schleef M., Schmeer M., Einsele H., Ivics Z., Hudecek M. (2017). Enhanced CAR T-cell engineering using non-viral Sleeping Beauty transposition from minicircle vectors. Leukemia.

[108] Kesselring L., Miskey C., Zuliani C., Querques I., Kapitonov V., Laukó A., Fehér A., Palazzo A., Diem T., Lustig J. (2020). A single amino acid switch converts the Sleeping Beauty transposase into an efficient unidirectional excisionase with utility in stem cell reprogramming. Nucleic Acids Res..

[109] Kebriaei P., Izsvák Z., Narayanavari S.A., Singh H., Ivics Z. (2017). Gene Therapy with the Sleeping Beauty Transposon System. Trends Genet..

[110] Kettlun C., Galvan D.L., George A.L., Kaja A., Wilson M.H. (2011). Manipulating piggyBac transposon chromosomal integration site selection in human cells. Mol. Ther..

[111] Yant S.R., Huang Y., Akache B., Kay M.A. (2007). Site-directed transposon integration in human cells. Nucleic Acids Res..

[112] Klompe S.E., Vo P.L.H., Halpin-Healy T.S., Sternberg S.H. (2019). Transposon-encoded CRISPR-Cas systems direct RNA-guided DNA integration. Nature.

[113] Strecker J., Ladha A., Gardner Z., Schmid-Burgk J.L., Makarova K.S., Koonin E.V., Zhang F. (2019). RNA-guided DNA insertion with CRISPR-associated transposases. Science.

[114] Jinek M., Chylinski K., Fonfara I., Hauer M., Doudna J.A., Charpentier E. (2012). A programmable dual-RNA-guided DNA endonuclease in adaptive bacterial immunity. Science.

[115] Kovač A., Miskey C., Menzel M., Grueso E., Gogol-Döring A., Ivics Z. (2020). RNA-guided retargeting of Sleeping Beauty transposition in human cells. ELife.

